# Dynamic Kinetics of MgH_2_ in Solid-State Hydrogen Storage: From the “Dam-Break Effect” to Mechanistic Understanding, Data-Driven Design, and Application Strategies

**DOI:** 10.1021/acs.accounts.6c00383

**Published:** 2026-07-14

**Authors:** Jianghao Cai, Tongao Yao, Piao Ma, Wenfeng Fu, Shuai Dong, Di Zhang, Linda Zhang, Zhengyang Gao, Hao Li, Weijie Yang

**Affiliations:** † School of Energy, Power and Mechanical Engineering, Department of Power Engineering, 91630 North China Electric Power University, Baoding 071003, China; ‡ Gusu Laboratory of Materials, Suzhou 215000, China; § Suzhou MatSource Technology Co., Ltd., Suzhou 215000, China; ∥ Advanced Institute for Materials Research (WPI-AIMR), Tohoku University, Sendai 980-8577, Japan; ⊥ Frontier Research Institute for Interdisciplinary Sciences, Tohoku University, Sendai 980-8577, Japan; # Hebei Key Laboratory of Energy Storage Technology and Integrated Energy Utilization, North China Electric Power University, Baoding 071003, China

## Abstract

Gas–solid reaction kinetics describes how gaseous species interact with solid surfaces and bulk regions in energy and materials processes. This behavior is often evaluated using compact descriptors, such as onset temperature, apparent activation energy, equilibrium parameters, or rate constants. These descriptors enable comparison across materials and conditions, but compress a multistage reaction into single-value metrics. The central limitation is that kinetic resistance is treated as an averaged parameter, rather than as a quantity that may emerge at a specific stage, location, or interface and evolve during conversion. This compression becomes problematic when local coordination, bonding, defect structure, heat transfer, mass transport, and interfaces change. MgH_2_ dehydrogenation provides a representative case. Its sluggish dehydrogenation has been assessed using onset dehydrogenation temperature and apparent activation energy. However, the weak correspondence between these parameters, the gap between theoretical barriers and experimentally fitted activation energy, and the ability of surface catalysts to improve macroscopic kinetics point to a missing kinetic coordinate. This coordinate is reaction progress, which reveals how much reaction has occurred, where dominant resistance is located, and how it is redistributed.

In this Account, we discuss the “dam-break effect” (originally proposed as the “burst effect”) as a reaction progress dependent kinetic framework for MgH_2_ dehydrogenation. Unlike conventional single-parameter descriptions, the dam-break effect emphasizes stage-resolved evolution of kinetic resistance from an initially high-resistance surface state to a lower-resistance regime. Microscopic calculations show that hydrogen removal from the first surface layer has the highest barrier, whereas subsequent hydrogen desorption and migration become easier after this layer is breached. Reconstruction of isothermal kinetic curves reveals a rapid decrease in activation energy in the early region, followed by a stable plateau. These observations suggest that the first surface layer behaves as a kinetic dam, and its removal triggers a transition from an initially resistant state to an accelerated regime. We then examine how the dam-break effect connects local environment evolution, surface-controlled dehydrogenation, and inconsistencies among onset temperature, apparent activation energy, and theoretical barriers. This dynamic viewpoint is extended to data-driven design, where databases, machine learning, AI agents, and machine learning potentials transform kinetic observations into computable descriptors. Finally, we discuss application strategies in which materials design targets the surface and near-surface bottleneck, while system operation matches energy input to stage-dependent resistance. Using MgH_2_ as a model system, this Account aims to promote a broader shift in gas–solid reaction kinetics from static parameter description toward reaction progress resolved dynamic interpretation.

## Graphical abstract

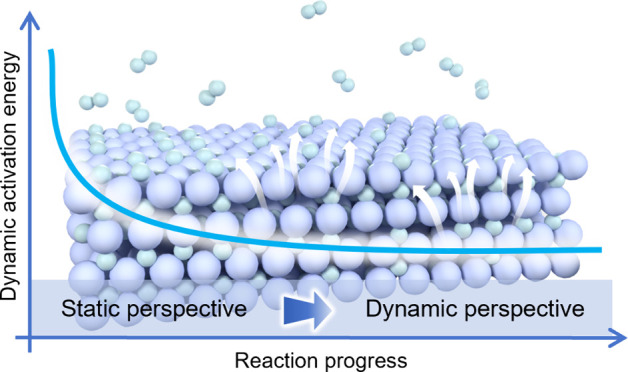

## Key References

-

Dong, S. ; Li, C. ; Wang, J. ; Liu, H. ; Ding, Z. ; Gao, Z. ; Yang, W. ; Lv, W. ; Wei, L. ; Wu, Y. ; Li, H. The “burst effect” of hydrogen desorption in MgH_2_ dehydrogenation. J. Mater. Chem. A 2022, 10, 22363–22372 10.1039/D2TA06458H .^1^ This paper first proposed the “dam-break effect” in MgH_2_ dehydrogenation, revealing that the first surface hydrogen desorption step acts as a kinetic dam whose breakthrough triggers a rapid decrease in dehydrogenation resistance.
-

Li, C. ; Yang, W. ; Liu, H. ; Liu, X. ; Xing, X. ; Gao, Z. ; Dong, S. ; Li, H. Picturing the Gap Between the Performance and US-DOE’s Hydrogen Storage Target: A Data-Driven Model for MgH_2_ Dehydrogenation. Angew. Chem., Int. Ed. 2024, 63­(28), e202320151 10.1002/anie.202320151 38665013.^2^ This paper built on the dam-break effect in MgH_2_ dehydrogenation and developed a general descriptor for single atom catalyst regulated MgH_2_ surfaces, linking interfacial electronic structure to dehydrogenation barrier prediction and catalyst screening.
-

Cai, J. ; Wang, H. ; Tang, X. ; Miao, Z. ; Yao, T. ; Liu, Y. ; Wang, H. ; Gao, Z. ; Yang, W. Data-driven evidence for the burst effect in MgH_2_ dehydrogenation via analysis of experimental kinetic curves. J. Energy Storage 2025, 136, 118450 10.1016/j.est.2025.118450 .^3^ This paper provided experimental kinetic evidence for the dam-break effect by reconstructing reaction progress dependent activation energy profiles from isothermal MgH_2_ dehydrogenation curves across pure, catalyst-modified, and alloyed systems.
-

Sato, R. ; Conway, L. J. ; Zhang, D. ; Pickard, C. J. ; Akagi, K. ; Sau, K. ; Li, H. ; Orimo, S. Surface melting-driven hydrogen absorption for high-pressure polyhydride synthesis. Proc. Natl. Acad. Sci. U.S.A. 2025, 122­(22), e2413480122 10.1073/pnas.2413480122 40440065 PMC12146707.^4^ This paper develops machine learning potentials to observe a dam-break-like hydrogenation process, showing that surface melting can overcome the initial kinetic threshold and trigger rapid hydrogen uptake under high pressure.

## 1 Introduction

Gas–solid reactions are central to energy storage, chemical engineering, environmental science, catalysis, metallurgy, and materials processing. They represent a fundamental class of processes that strongly influence material reactivity and practical performance.^5–7^ These reactions usually involve chemical transformations between gaseous species and solid surfaces or solid interiors, together with coupled heat and mass transfer and the continuous evolution of the interface. Their kinetic behavior directly determines reaction rates, conversion efficiency, and 000process regulation. As a result, gas–solid reaction kinetics has remained a central topic in related fields (Figure 1a).^8,9^ Hydrogen storage is one of the most important application scenarios in which gas–solid reactions play a decisive role.^4,7^ Among the many representative gas–solid reaction systems, the dehydrogenation of MgH_2_ has attracted sustained attention because of its well-defined material basis and strong application relevance.^1–3,10^ As a high-capacity solid-state hydrogen storage material, MgH_2_ offers high theoretical hydrogen storage density and good resource availability, which makes it one of the most promising hydrogen storage systems.^11,12^

**Figure 1:**
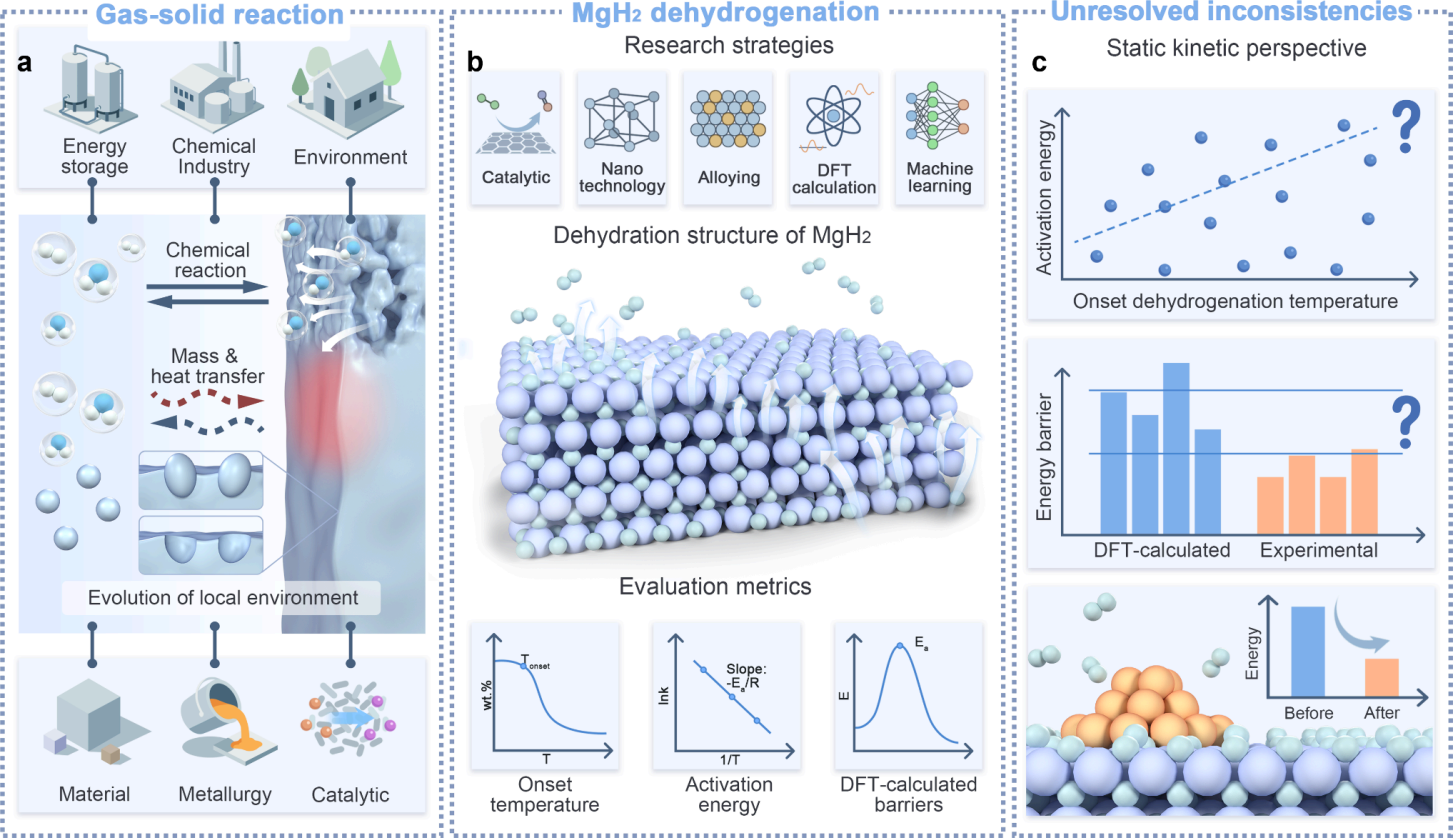
Gas–solid reaction background and key issues in MgH_2_ dehydrogenation. (a) General schematic of gas–solid reactions and their broad application fields. (b) Key research directions and major evaluation metrics in MgH_2_ dehydrogenation. (c) Principal unresolved issues in current MgH_2_ dehydrogenation studies, including unclear descriptor correlations, gaps between theoretical and experimental kinetic parameters, and insufficient connections between surface regulation and macroscopic dehydrogenation behavior.

Despite these advantages, the practical application of MgH_2_ has long been limited by sluggish dehydrogenation kinetics. To address this issue, extensive efforts have been devoted to material modification. For example, catalytic components such as transition metals,^13^ metal oxides,^14^ and carbon-based materials^15,16^ have been introduced to improve reaction activity. Nanostructuring, particle refinement, and size control have also been used to shorten diffusion pathways.^17^ In addition, alloying, compositing, and interfacial structure engineering have been employed to regulate the local reaction environment and dehydrogenation behavior of the material.^18^ On this basis, the kinetic performance of MgH_2_ is commonly evaluated using descriptors such as the onset dehydrogenation temperature and the apparent activation energy. The onset dehydrogenation temperature is often used to reflect how readily hydrogen release begins, whereas the apparent activation energy is commonly used to characterize the overall kinetic barrier of the dehydrogenation process. Together, these studies have continuously advanced the understanding of MgH_2_ dehydrogenation from the perspectives of material design and kinetic evaluation. They also define the main framework that has guided research in this field to date (Figure 1b).^19,20^

However, three principal contradictions remain unresolved in current studies. First, no clear correspondence has been established between the onset dehydrogenation temperature and the apparent activation energy.^21–27^ Second, a pronounced gap has long persisted between the energy barrier obtained from theoretical calculation and the apparent activation energy derived from experimental fitting.^3,28–30^ Third, catalysts often act mainly at the material surface, yet they can still improve the overall dehydrogenation kinetics.^25,31,32^ These observations suggest that conventional kinetic descriptions lack a critical dimension, namely reaction progress, and therefore cannot fully reveal the kinetic nature of MgH_2_ dehydrogenation (Figure 1c). In this Account, we shift from static parameter-based evaluation to reaction progress dependent dynamic characterization. Centered on the evolution of reaction resistance, we introduce the dam-break effect and discuss its implications for mechanistic understanding, data-driven design, and application strategies. Through this framework, we aim to provide a new basis for understanding MgH_2_ dehydrogenation and for moving gas–solid reaction kinetics from static parameter description toward process-evolution-based interpretation.

## 2 Dam-Break Effect

Previous studies have shown that MgH_2_ dehydrogenation is not governed by a single elementary step, but involves several coupled events, including surface hydrogen desorption, bulk hydrogen migration, and continuous reorganization of local bonding states.^33–35^ To clarify this microscopic process, a stable multilayer MgH_2_ surface model was constructed, and stepwise dehydrogenation from the surface toward the bulk was systematically calculated using the climbing-image nudged elastic band (CI-NEB) method (Figure 2a).^1^ The results show that hydrogen desorption from the first surface layer corresponds to the highest kinetic barrier in the entire dehydrogenation process, about 2.52–2.53 eV. After first-layer hydrogen removal, subsequent desorption barriers decrease markedly to 1.14–1.39 eV, while hydrogen migration barriers are generally lower (Figure 2b).^1^ This phenomenon is designated the “dam-break effect” (originally proposed as the “burst effect”). Bonding analysis indicates that Mg–H bonds in the reaction region gradually weaken as dehydrogenation proceeds, thereby facilitating later hydrogen migration and desorption. Electronic structure analysis further shows that strong electron localization is retained near the surface before first-layer hydrogen removal, which explains the high initial kinetic resistance. Consistently, ab initio molecular dynamics (AIMD) simulations indicate that bulk hydrogen mobility becomes enhanced only after sufficient surface hydrogen removal, providing microscopic support for the subsequent acceleration of dehydrogenation.

**Figure 2:**
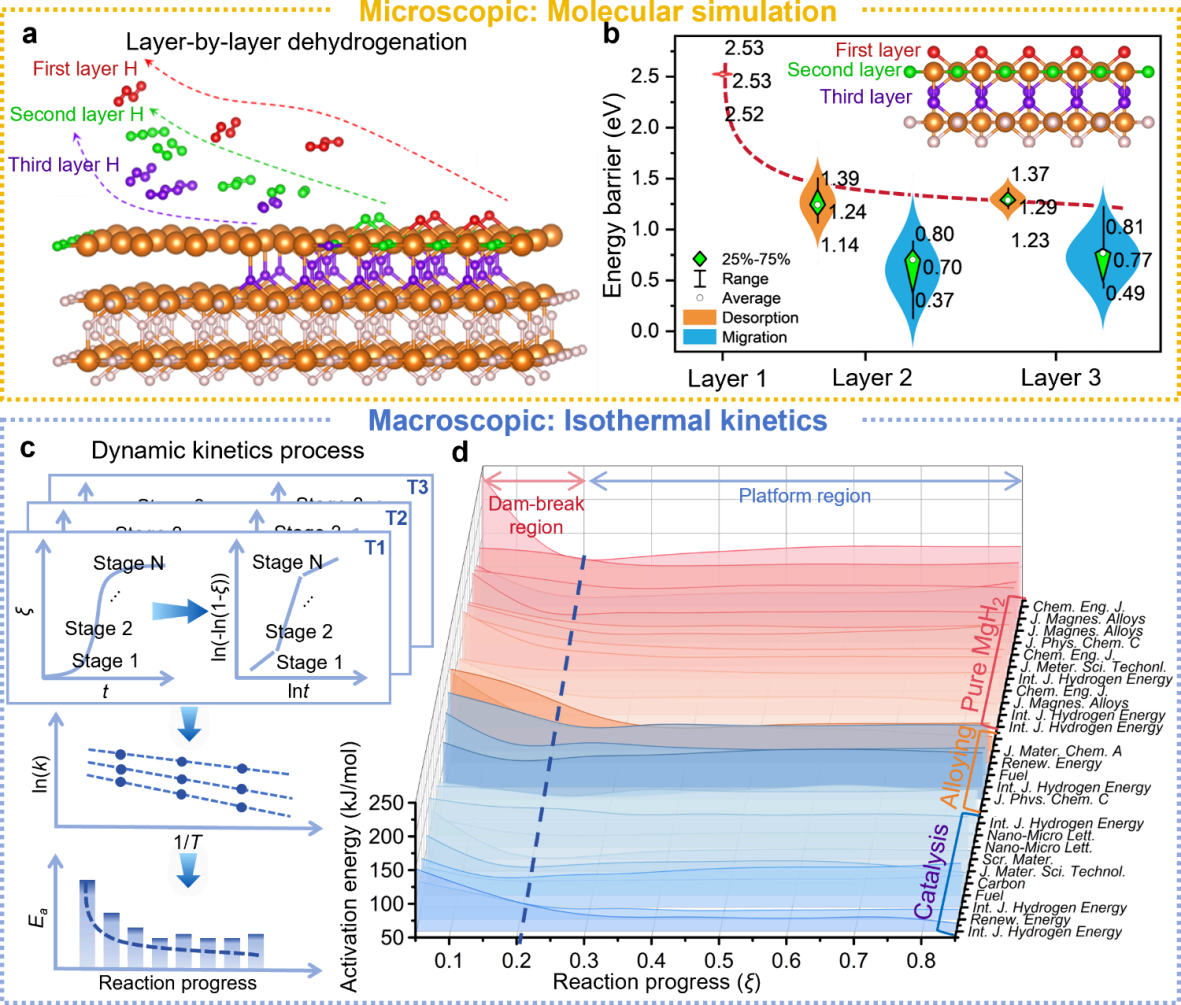
Microscopic and macroscopic evidence for the dam-break effect in MgH_2_ dehydrogenation. (a) Schematic illustration of layer-by-layer dehydrogenation in MgH_2_. (b) Calculated energy barriers for hydrogen desorption and hydrogen migration at different dehydrogenation layers in MgH_2_. (c) Workflow for the dynamic kinetic analysis of isothermal MgH_2_ dehydrogenation. (d) Statistical summary of the dynamically processed isothermal dehydrogenation kinetic curves reported for pure MgH_2_, catalyst-modified Mg-based systems, and alloyed Mg-based systems. [Panels (a,b) were adapted from ref 1, copyright 2022 Royal Society of Chemistry. Panels (c,d) were adapted from ref 3, copyright 2025 Elsevier.]

On this basis, isothermal dehydrogenation kinetic curves for pure MgH_2_, catalyst-modified Mg-based systems, and alloyed Mg-based systems were dynamically analyzed using the Johnson-Mehl-Avrami–Kolmogorov (JMAK) kinetic equation and the Arrhenius relationship (Figure 2c).^36,37^ This analysis yielded activation energy profiles that evolve continuously with reaction progress (ξ) (Figure 2d).^3^ A common pattern appears across these systems: the activation energy decreases sharply at the initial stage and then evolves into a more gradual plateau-like regime. The initial interval with the most pronounced activation-energy decrease, usually within approximately 10–20% reaction progress, is defined as the dam-break region. The subsequent broader interval, in which the activation energy changes more slowly and remains relatively stable, is defined as the plateau region. The repeated appearance of these two regions in macroscopic kinetic data sets suggests that resistance release is not an isolated observation, but a recurring kinetic feature of MgH_2_ dehydrogenation.^3^

The microscopic layer-by-layer mechanism and the macroscopic kinetic statistics show that MgH_2_ dehydrogenation is not a uniform process governed by the same resistance throughout the reaction. Instead, it evolves from an initially high-resistance stage to a stage of rapid barrier reduction and then to a relatively stable stage. This physical picture resembles the resistance change before and after the breaching of a dam. The high barrier associated with first-layer hydrogen desorption acts like a dam blocking hydrogen release. Once this barrier is overcome, the barriers for subsequent hydrogen migration and desorption decrease significantly, and the overall kinetic resistance drops rapidly. This stage-dependent kinetic behavior can be described as the dam-break effect in MgH_2_ dehydrogenation.^1,31^ In this framework, the high resistance of the first layer at the atomic scale is directly linked to the parameter evolution observed in macroscopic kinetics, indicating that MgH_2_ dehydrogenation should be understood as a stage-dependent process in which reaction resistance continuously evolves with reaction progress.

## 3 Mechanistic Understanding

### 3.1 Evolution of the Local Environment

MgH_2_ dehydrogenation does not proceed in a fixed local environment. Instead, it involves continuous changes in coordination, bonding, and defect structures. As Mg–H bonds are progressively broken, the original coordination environment around hydrogen is disrupted, and local bond lengths, bond strengths, and charge distributions continuously evolve. Hydrogen vacancy formation, local relaxation, and structural rearrangement therefore persist throughout dehydrogenation.^38,39^ From this perspective, the microscopic dehydrogenation pathway of MgH_2_ can be divided into four consecutive stages, namely first-layer hydrogen desorption, bulk hydrogen migration, surface hydrogen diffusion, and continuous surface hydrogen desorption (Figure 3a). First-layer hydrogen desorption corresponds to the high-barrier stage and determines the difficulty of reaction initiation, whereas the subsequent stages are associated with overall lower barriers. As the reaction proceeds inward from the surface, the local environment evolves from a relatively complete and stable bulk coordination state into a near-surface environment characterized by abundant vacancies, reduced coordination, weakened structural constraints, and more favorable conditions for hydrogen migration and desorption.

**Figure 3:**
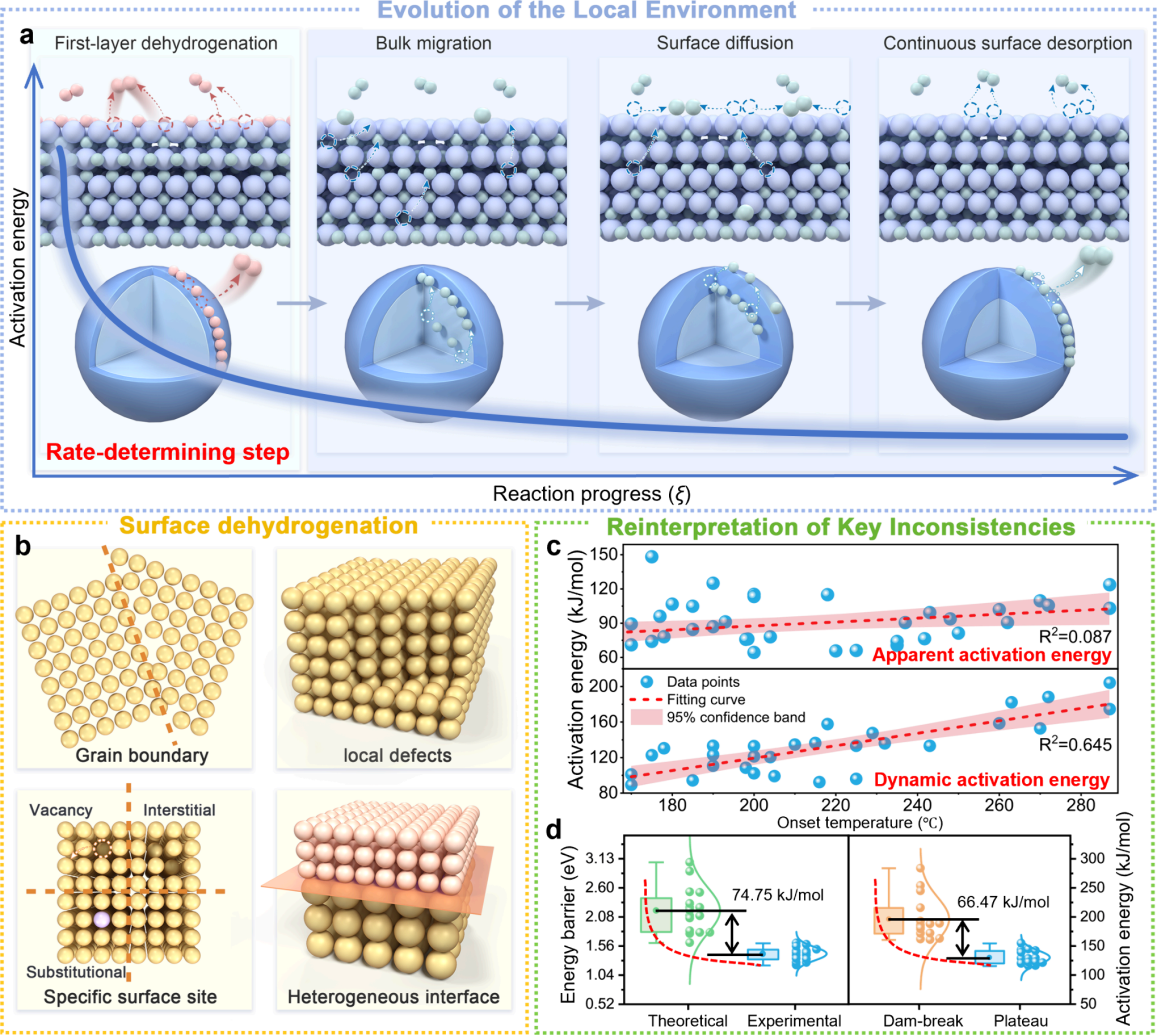
Mechanistic interpretation of stage-dependent dehydrogenation in MgH_2_. (a) Schematic illustration of the microscopic dehydrogenation pathway in MgH_2_, including first-layer hydrogen desorption, bulk hydrogen migration, surface hydrogen diffusion, and continuous surface hydrogen desorption, together with the corresponding evolution of energy barriers. (b) Key factors affecting the local surface environment during MgH_2_ dehydrogenation. (c) Statistical correlations among the initial surface dehydrogenation activation energy, the apparent dehydrogenation activation energy, and the onset dehydrogenation temperature. (d) Statistical relationship between theoretical energy barrier and experimentally fitted activation energy. [Panel (d) was adapted from ref 3, copyright 2025 Elsevier.]

This picture is supported by both experiment and theory. *In situ* characterization shows that MgH_2_ does not transform uniformly at the early stage of dehydrogenation. Instead, the Mg phase first appears at the top interface, accompanied by void and crack formation, and the transformation front then propagates downward.^40^ The MgH_2_/Mg transformation also involves nucleation and growth, with different grain-growth modes at different stages.^41^ In nanoconfined and *in situ* catalytic systems, the multiphase interface between MgH_2_/Mg and Ti-MX, especially the *in situ* formed TiH_2_ interface, further modifies the local structure near the interface.^42^ Theoretical calculations likewise show that changes in the local environment alter the barriers of elementary processes, including hydrogen vacancy formation, migration of hydrogen related defects, and Mg–H bond cleavage.^43–45^ The continuous evolution of the microscopic local environment from a fully coordinated state to a defect-rich, low-coordination state is therefore not merely a consequence of reaction progress, but the fundamental origin of the changing difficulty and control relationships among the elementary steps. At the macroscopic level, the evolution of apparent activation energy and related kinetic parameters with reaction progress is an external manifestation of this persistent local environment reconstruction.

### 3.2 Surface Dehydrogenation and Influencing Factors

Although MgH_2_ dehydrogenation involves continuous local environment evolution, desorption of hydrogen from the first surface layer still determines whether the reaction can be effectively initiated and therefore controls the onset of dehydrogenation. Surface hydrogen vacancy formation and first-layer hydrogen release are highly sensitive to crystal orientation and surface stability, and large differences in vacancy formation energies and dehydrogenation difficulty have been reported among crystal facets. A more stable initial surface generally requires a higher barrier for first-layer hydrogen removal, which corresponds to the sluggish dehydrogenation kinetics observed at the early stage.^46^ Mechanistic calculations further show that surface dehydrogenation of MgH_2_ consists of two consecutive processes, Mg–H bond cleavage and H_2_ desorption, with Mg–H bond cleavage serving as the key step that governs the difficulty of surface hydrogen removal.^47^ Even on defective MgH_2_(110) surfaces containing vacancies, the barrier of this step can be significantly reduced, yet it still remains the principal kinetic hurdle at the initial stage.^48^ These results indicate that first-layer hydrogen desorption is not merely an initiating event, but the high-barrier step that must be crossed for MgH_2_ dehydrogenation to proceed continuously (Figure 3a).

Grain boundaries, local defects, special surface sites, and heterogeneous interfaces may all promote surface dehydrogenation by modifying the near-surface local environment (Figure 3b). During early MgH_2_ decomposition, hydrogen vacancies tend to become ordered and gradually form an Mg(0001)/MgH_2_(110) interface, which weakens Mg–H bonds and facilitates both hydrogen removal and migration near the interface.^49^ *In situ* observations further reveal that hydride transformation preferentially nucleates at grain boundaries and then propagates across grains, whereas first-principles calculations show that different grain-boundary configurations exhibit markedly different H_2_ dissociation activities, with some grain-boundary sites displaying much lower dissociation barriers than Mg(0001).^50,51^ Surface catalytic regulation also mainly acts on near-surface processes, including H_2_ adsorption and dissociation, surface penetration, and heterogeneous nucleation, while newly formed surface active phases generated by atomic reconstruction can further weaken Mg–H bonds and reduce the migration barrier of hydrogen near the surface.^52^ However, these factors mainly facilitate near-surface dehydrogenation rather than fundamentally altering the lower intrinsic barriers for bulk hydrogen diffusion and migration. Surface layer dehydrogenation therefore remains the primary kinetic obstacle along the overall pathway and continues to occupy the rate-determining step in MgH_2_ dehydrogenation.

### 3.3 Reinterpretation of Key Inconsistencies

The onset dehydrogenation temperature and the apparent activation energy probe different kinetic regimes in MgH_2_ dehydrogenation. The onset temperature is governed by the most difficult event at the beginning of dehydrogenation and therefore primarily reflects the initial high-barrier process associated with first-layer hydrogen desorption.^45,46^ In contrast, the apparent activation energy is usually extracted from the quasi-steady portion of isothermal dehydrogenation curves and is weighted more strongly by sustained dehydrogenation over a broader conversion range, making it more representative of the average kinetic level of the later plateau regime.^40^ This distinction becomes clear after progress-resolved reconstruction of literature isothermal dehydrogenation curves. The reported apparent activation energy shows only a very weak correlation with onset dehydrogenation temperature, with a correlation coefficient of 0.087, whereas the initial dehydrogenation activation energy shows a much stronger correlation, reaching 0.645 (Figure 3c). These results indicate that onset behavior is controlled not by the average barrier of the later quasi-steady stage, but by the initial high barrier that governs reaction initiation. The long-recognized inconsistency between onset dehydrogenation temperature and apparent activation energy therefore arises because the two quantities reflect different segments of the dehydrogenation process.

The discrepancy between activation energy from theoretical calculations and experimental fitting should likewise be interpreted within a multistage kinetic framework. Most theoretical studies focus on surface or early stage microscopic events, such as surface hydrogen vacancy formation, first-layer hydrogen desorption, defect-site dehydrogenation, and interfacial hydrogen migration.^48–55^ These calculations capture the difficult elementary barriers associated with initial local activation and therefore correspond more closely to the high-barrier region at the beginning of the dynamic activation energy profile.^3,31^ By contrast, apparent activation energy from experimental fitting reflects the integrated response of macroscopic kinetic curves and is more representative of the later quasi-steady stage.^31^ Statistical analysis supports this interpretation. The mean difference between activation energy from theoretical calculations and experimental fitting is 74.75 kJ mol^–1^, close to the 66.47 kJ mol^–1^ mean difference between the dam-break and plateau regions in the dynamic activation energy profile (Figure 3d).^3^ This agreement indicates that theoretical and experimental values are not necessarily conflicting, but may represent barrier information from different stages of the same dehydrogenation process. Within the dam-break effect framework, MgH_2_ dehydrogenation is therefore better understood as a continuous multistage kinetic evolution from an initial high-resistance surface-activation stage to a later relatively stable dehydrogenation stage, in which local environment evolution, surface-controlled hydrogen release, and the dynamic variation of apparent activation energy are coupled within the same reaction process.

## 4 Data-Driven Design

### 4.1 Hydrogen Storage Materials Database and Machine Learning

The dam-break effect indicates that MgH_2_ dehydrogenation is not a uniform process governed by a single static activation energy, but a dynamic process that evolves continuously with reaction progress. From this perspective, kinetic data should no longer be recorded only as isolated onset temperatures or apparent activation energy. Instead, they should be organized according to material composition, treatment strategy, testing condition, and reaction stage. Accordingly, databases are no longer simple archives of literature data, but the foundation for unified and scalable studies of dynamic kinetics. Data platforms represented by *Digital Hydrogen-S* (http://digital-hydrogen.com/storage/index.html) and Digital Hydrogen Platform (*DigHyd*: https://www.dighyd.org) have begun to integrate material composition, structural information, thermodynamic and kinetic parameters, and testing conditions that were previously scattered across the literature into structured data resources (Figure 4a). *Digital Hydrogen-S Digital Hydrogen-S* integrates standardized solid-state hydrogen storage data for more than 3,000 materials, including PCT and kinetic curves, interactive benchmarking, and AI-assisted analysis,^56^ whereas *DigHyd* consolidates more than 30,000 entries from over 4,000 publications.^57,58^ On this basis, machine learning models can identify the factors governing dehydrogenation behavior from large scale data sets and enable performance prediction, efficient screening, and design optimization. When further combined with agent-based design, this framework can connect databases, feature extraction, model prediction, and candidate recommendation, thereby moving MgH_2_ dehydrogenation research from empirical exploration toward intelligent design (Figure 4b).

**Figure 4:**
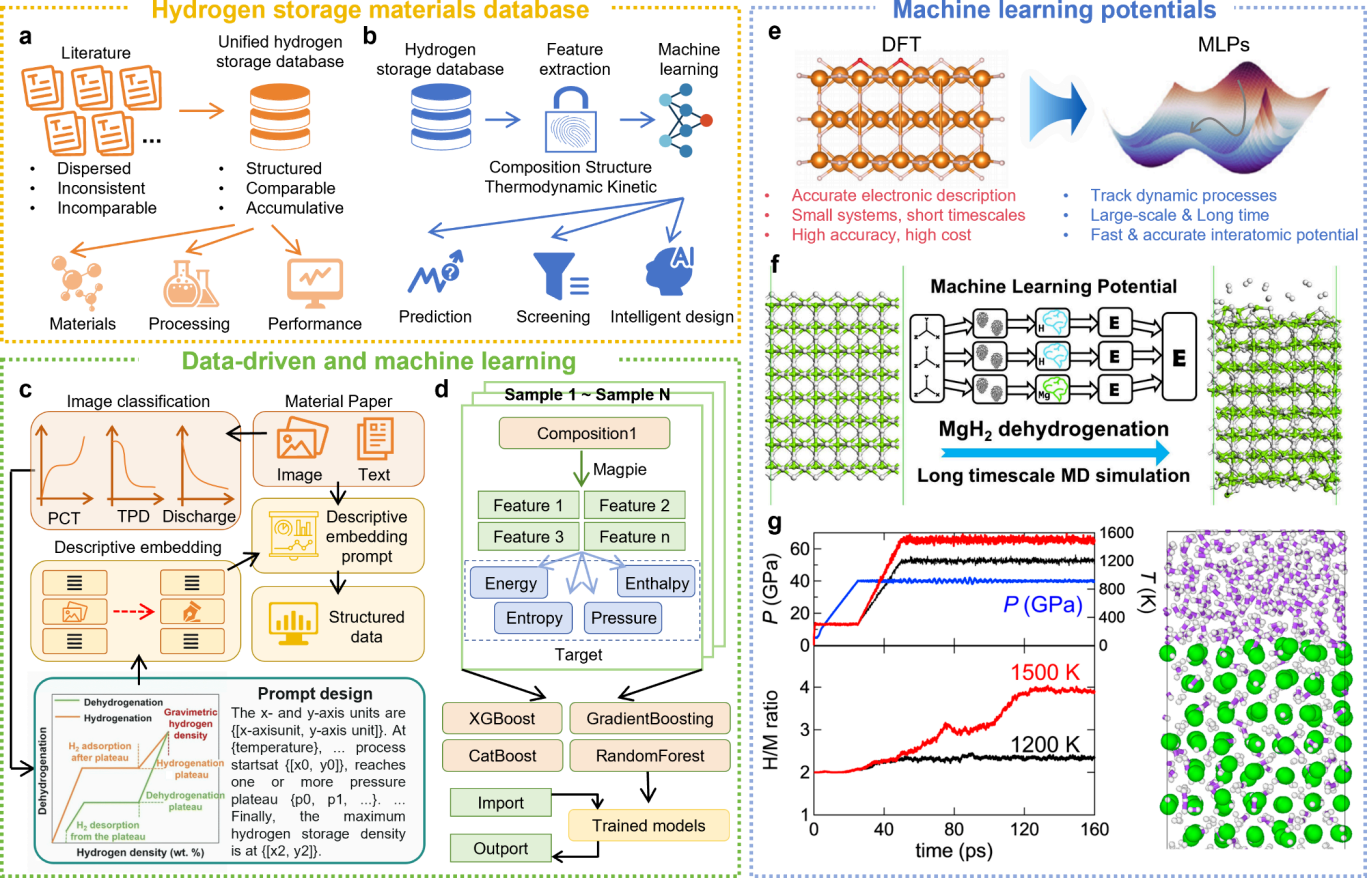
Data-driven workflows and machine learning potentials for dynamic hydrogen storage research. (a) Transformation of dispersed literature information into a unified hydrogen storage materials database. (b) Conversion of structured hydrogen storage databases into feature representations for machine learning. (c) Workflow of the Descriptive Interpretation of Visual Expressions (DIVE). (d) Data-driven research pathway from material information to feature extraction, model training and predictive model construction. (e) Progressive extension from first-principles calculations to machine learning potentials for larger scale and longer time scale dynamic simulations. (f) MLP framework enabling long-time scale molecular dynamics simulations of MgH_2_ dehydrogenation. (g) (left) Snapshots of atomic configurations obtained during molecular dynamics simulations of the CaH_2_(100)/H_2_ interface at 1500 K. (right) Time series of temperature, pressure, and H/M ratio from MLP-MD simulations of the CaH_2_(100)/H_2_ interface at 40 GPa. [Panel (c) was adapted from ref 58, copyright 2026 Royal Society of Chemistry. Panel (f) was reproduced from ref 70, copyright 2025 American Chemical Society. Panel (g) was reproduced from ref 4, copyright 2025 National Academy of Sciences.]

More importantly, such databases make cross-system feature extraction possible, allowing mechanistic insights such as local environment evolution, surface control, and dynamic activation energy to be translated into descriptors suitable for comparison, representation, and modeling. The multiagent workflow demonstrated by the Descriptive Interpretation of Visual Expression (DIVE) shows that experimental information embedded in figures and tables can be automatically extracted, organized, and converted into structured knowledge for inverse design, bringing previously scattered material information into a unified framework for data representation and knowledge organization (Figure 4c).^58,59^ Studies integrating databases, forward prediction, and inverse design into a single framework, as illustrated by the *FIND* platform for hydrogen storage alloys (https://find4hyalloy.zicp.fun/), show that data-driven research is moving beyond fitting accuracy toward transferable and navigable materials design frameworks.^60–63^ The combination of large language models with machine learning is further connecting data extraction, knowledge organization, performance prediction, and design recommendation generation, making it possible to integrate database construction, model training, and candidate recommendation within a unified workflow.^64–66^ The *Cat-MH* platform (https://cat-metalhy.cpolar.top/) for metal hydrides provides a representative example of this emerging direction.^60,67^ These advances define a clear data-driven route in which feature information is extracted from material information, followed by model training, predictive model construction, and subsequent use in performance prediction, candidate screening, and design applications (Figure 4d).^56–69^ In addition, physically interpretable descriptor models built on large scale databases show that data-driven research does not have to remain at the level of black-box prediction, but can also yield design maps and material selection rules with clear physical meaning.^57^ Within the framework of the dam-break effect, the dynamic kinetic perspective has therefore evolved from a purely mechanistic issue into a data problem, and further into a route from databases to computable descriptor construction, label definition, and then to machine learning driven prediction, screening, and agent-based design of materials.

### 4.2 Machine Learning Potentials (MLPs) for Hydrogen Absorption and Desorption

MLPs provide a new technical foundation for atomistic characterization of dynamic hydrogen storage and release in Mg-based materials. The key implication of the dam-break effect lies not in a single barrier value itself, but in the stage transition from an initially high resistance regime to a subsequently accelerated regime, which can be described by layer-dependent barriers, local coordination environments, and resistance evolution with reaction progress. Conventional DFT calculations are effective for identifying local reaction pathways and key barriers, yet they are usually unable to directly capture the full dynamic evolution because of limitations in system size and time scale.^31,50^ MLPs can extend molecular dynamics simulations to much larger systems and longer time scales while retaining near first-principles accuracy. This capability shifts dynamic kinetic studies from static barrier analysis toward direct observation of continuous reaction trajectories (Figure 4e). Existing studies on Mg–H systems have already demonstrated the promise of this approach. Early work constructed MLPs for MgH_2_ nanoclusters, showing that the method can address nanoscale structures and kinetic processes that are difficult to access with *ab initio* calculations.^68^ Later long-time simulations of MgH_2_ dehydrogenation directly captured the formation of subsurface H_2_ and its retention in the near surface region, providing a new dynamic interpretation of the sluggish dehydrogenation behavior of MgH_2_ (Figure 4f).^70^

MLPs also show a clear advantage in resolving continuous kinetic processes on the hydrogenation side. Comparative studies of hydrogen diffusion in magnesium have shown that MLPs developed or refined through active learning can reproduce diffusion coefficients and activation energy with good accuracy, indicating that migration processes relevant to hydrogen uptake can be described more reliably at the atomic scale within a continuous kinetic framework.^71^ A related example comes from hydrogen uptake in CaH_2_ under high pressure, where MLP-based molecular dynamics directly showed that hydrogenation begins with surface melting and then proceeds rapidly through a liquid-like CaH_4_ intermediate (Figure 4g).^4^ The surface is activated first, the hydrogen content then rises quickly, and the local structure evolves toward a state more favorable for hydrogen uptake. This behavior indicates a stage transition controlled by an initial kinetic threshold rather than a uniform process. Thermodynamic analysis further showed that high pressure both favors the hydrogenation enthalpy and lowers the energy required to form the liquid-like intermediate, making this pathway kinetically more accessible. Although this study focused on high-pressure CaH_2_ polyhydrides rather than MgH_2_, it provides a useful mechanistic analogy for understanding how an initial kinetic threshold can trigger rapid hydrogen uptake in metal hydride systems. MLPs may therefore help extend dynamic kinetic studies from dehydrogenation to hydrogenation and support a more unified description of hydrogen storage and release in metal hydride systems, thereby linking the dam-break effect to computable microscopic descriptors for data-driven design.

## 5 Application Strategies

### 5.1 Materials Design

Within the dam-break framework, the primary target for MgH_2_ materials design is the surface dehydrogenation step, which remains rate-determining and carries a higher barrier than bulk hydrogen migration and diffusion. The surface and near-surface regions therefore define the key reaction domain governing both the onset difficulty and the overall progression of dehydrogenation (Figure 5a). Recent experiments have converged on this point. Single-atom Ni on TiO_2_,^72^ single-atom Cu on Ti_3_C_2_T_*x*_,^73^ and Ru single atoms on Nb_2_O_5_ differ in support and active-site structure,^74^ yet they all reduce the onset dehydrogenation temperature of MgH_2_ and improve hydrogen sorption kinetics through near-surface regulation mechanisms, including the creation of dispersed active sites, vacancy-rich environments, and electronically tunable interfacial centers (Figure 5b). Their shared effect is to facilitate surface Mg–H bond activation and the onset of the first dehydrogenation step, thereby accelerating the rate-determining step and improving the overall process. The Mn_1.48_Ti_1.1_V_0.3_Zr_0.12_ nanosheet system further introduced the concept of an enhanced dam-break effect, showing that multiphase interfaces and hydrogen pump effects can help the system overcome the initial high-resistance stage more effectively.^75^ Materials design should therefore shift from average property optimization to targeted optimization of the key reaction domain, with priority placed on directed modification of the surface and near-surface region.

**Figure 5:**
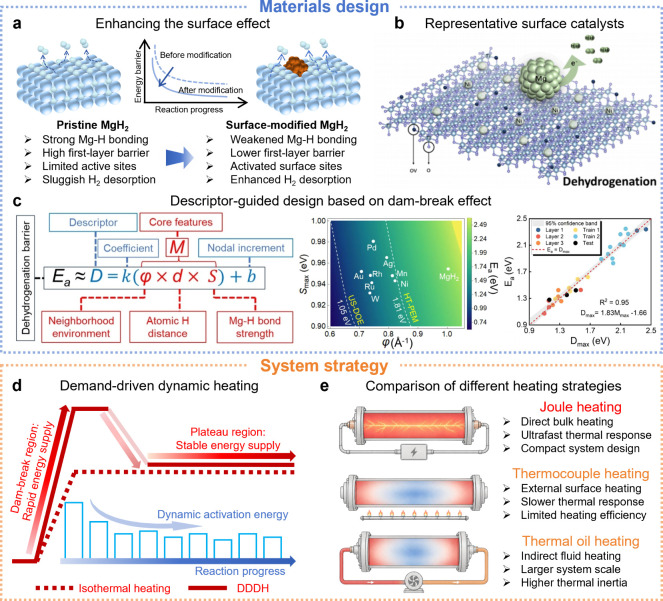
Materials-design and system-level strategies guided by the dam-break effect in MgH_2_ dehydrogenation. (a) Schematic illustration of surface modification of MgH_2_ and its promoting effect on the microscopic dehydrogenation process. (b) Mechanism of MgH_2_ dehydrogenation catalyzed by single-atom Ni supported on oxygen-vacancy-rich TiO_2_ (Ni@TiO_2_–OV). (c) (left) Descriptors that may affect MgH_2_ dehydrogenation kinetics. (middle) Derived contour plot predicting the dehydrogenation energy barrier of metal-doped MgH_2_ as a function of *S*_*max*_ and φ. (right) Correlation between the calculated activation energy and the descriptor *D*_*max*_. (d) Demand-driven dynamic heating (DDDH) strategy designed based on the dynamic activation energy evolution during MgH_2_ dehydrogenation. (e) Schematic comparison of Joule heating, external resistance heating, and heat transfer fluid heating. [Panel (b) was reproduced from ref 72, copyright 2024 American Chemical Society. Panel (c) was reproduced from ref 2, Copyright 2024 Wiley-VCH. Panel (d) was adapted from ref 85, copyright 2025 Elsevier.].

Theoretical studies further provide a clearer basis for stage-oriented design. Calculations on MgH_2_ supported single-atom catalyst heterojunctions and γ-graphdiyne supported single-atom catalysts show that isolated metal sites can markedly lower the surface dehydrogenation barrier, and that this reduction is closely tied to the interfacial electronic structure. Parameters such as system electronegativity and electrostatic potential can already be used to describe how different single-atom catalysts regulate the first surface dehydrogenation step.^76,77^ Microscopic scaling relations in Ti-based catalysts further show that changes in oxidation state within the same element can continuously tune Cat-H bond strength, thereby improving the balance between hydrogen dissociation and diffusion. This result suggests that materials design should move beyond simple elemental substitution and focus instead on precise control of the local electronic structure at the surface.^78,79^ More importantly, data-driven descriptors developed for MgH_2_ dehydrogenation have narrowed the key reaction domain even further to the surface and near-surface layers, and introduced a distance energy ratio descriptor defined by Mg–H bond COHP and the H–H atomic distance. This descriptor can predict dehydrogenation barriers at a computational cost far below that of conventional transition-state searches, providing a direct basis for catalyst screening and surface regulation (Figure 5c).^2^ Materials design should therefore shift from minimizing average activation energy to targeted optimization of the first surface dehydrogenation step, with emphasis on constructing more favorable interfacial electronic structures and surface-active sites around the true kinetic bottleneck.

### 5.2 System Strategy

System-level strategy should shift from improving average heat-transfer capacity under steady conditions to matching energy input with the stage-dependent evolution of dehydrogenation resistance. Recent studies on dual-reactor systems have coupled heat-transfer rate, circulating-fluid flow rate, and pressure difference within unified control frameworks,^80^ while temperature-driven coupled systems have integrated transient load, hydrogen transfer rate, and temperature and pressure responses into continuous regulation.^81^ Rapid and adjustable heating methods, including thermoelectric heat exchangers and electromagnetic induction heating, further indicate that responsive heat input is becoming important for reactor optimization.^82–84^ Within the dam-break framework, demand-driven dynamic heating provides a possible device-level route for stage-specific energy supply, in which heat input is adjusted according to reaction stage and resistance characteristics rather than maintained as a constant value (Figure 5d).^85^ Stronger and faster input may help overcome the initial high-resistance surface step, whereas lower-intensity sustaining input may be more suitable after the system enters later lower-resistance regimes.

External resistance heating and thermal oil heating are two common external energy-input modes in Mg-based hydrogen storage systems, yet both rely on gradual heat transfer from outside the reaction zone.^80–85^ They are therefore affected by heat conduction through the reactor wall and by the thermal inertia of external circulating units, which may limit rapid and concentrated energy delivery to the initial high-resistance surface stage. By comparison, Joule heating generates heat in a conductive component or conductive composite through resistive energy conversion, offering the potential for rapid temperature rise, controllable energy input, and a defined heat-generation location (Figure 5e). These features suggest a possible route for energy supply that is more closely matched to the stage-dependent kinetic demand of Mg-based hydrogen storage systems. In addition to possible field-related effects on electronic structure, phase behavior, and hydrogen diffusion pathways,^86^ Joule heating may provide localized resistive heating near the heating element or reaction zone, thereby improving transient thermal response and reducing ineffective heat-transfer loss.^87,88^ For MgH_2_ dehydrogenation, the initial surface dehydrogenation barrier is the highest, implying that rapid early stage heating may help overcome the first high-resistance step. After the system enters a later low-resistance stage, the energy-input intensity could potentially be reduced. Therefore, Joule heating and stage-matched energy supply should be regarded as potential design implications of the dam-break effect, rather than fully established engineering rules.

## 6 Extension and Generalization

The dam-break effect observed in MgH_2_ dehydrogenation provides direct evidence that this reaction cannot be adequately represented by a single activation energy, a single equilibrium parameter, or a single-stage rate expression, because the local environment, rate-controlling step, and kinetic resistance evolve as the reaction proceeds.^1–3^ For MgH_2_, reaction progress should therefore be treated not merely as a coordinate of conversion, but as an additional dimension for describing the evolution of physical parameters and process-dependent behaviors associated with local activation, resistance redistribution, and migration of the rate-determining step (Figure 6a). Beyond MgH_2_, we propose that this stage-resolved perspective may also offer a useful hypothesis for analyzing other gas–solid reactions, rather than a universal rule that has already been fully established. Related process-dependent phenomena have been reported in other systems. For example, interfacial initiation accompanied by stage switching has been observed in high pressure hydrogenation,^4^ and studies of gas–solid interface reactions have revealed localized initiation and interfacial structural adjustment.^5,39,89,90^ These observations suggest that broader gas–solid reactions may also involve progress-dependent changes in thermodynamic, kinetic, and transport parameters. In catalytic reactions, the peak position of volcano plots may shift as active sites evolve, surface intermediates accumulate, electronic structures change, and descriptor relations drift. In phase-change or thermochemical heat-storage processes, reaction enthalpy may vary as hydration interfaces advance, product layers thicken, pores become blocked, and structures reorganize. In diffusion-controlled processes, the diffusion coefficient may also evolve with pore-structure change, increasing transport tortuosity, product-layer densification, and progressive pathway blockage (Figure 6b). Thus, while the dam-break effect is directly supported by MgH_2_ evidence, its broader implication is that complex gas–solid reactions should be examined through reaction-progress-based dynamic process description. This stage-resolved perspective may provide a basis for developing more general analytical frameworks for gas–solid reaction kinetics.

**Figure 6:**
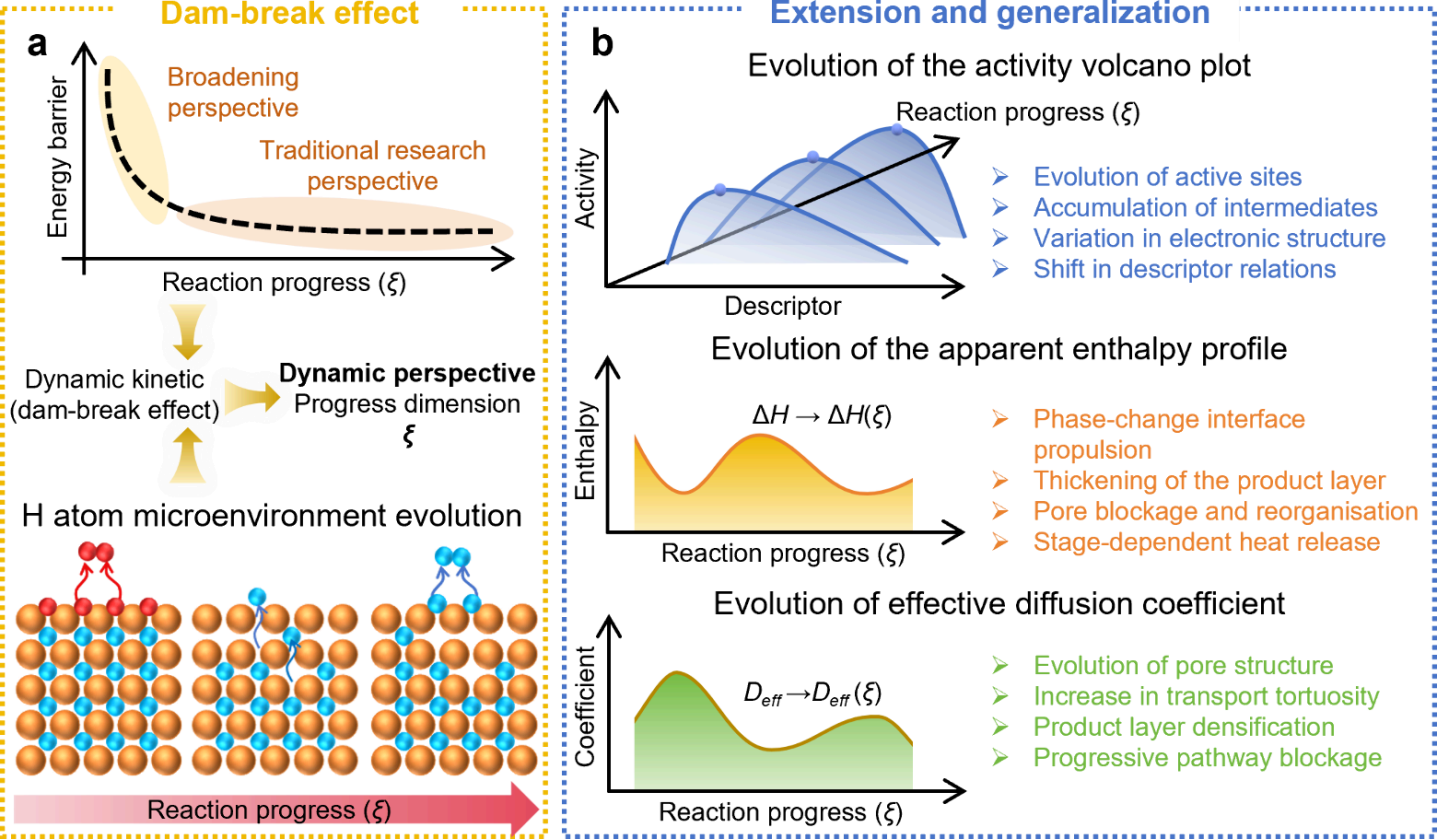
Reaction progress dimension derived from MgH_2_ dehydrogenation for broader gas–solid reaction kinetics. (a) From dynamic kinetics (dam-break effect) in MgH_2_ dehydrogenation to a reaction progress dimension for broader gas–solid reactions. (b) Potential dynamic characteristics in gas–solid reactions that evolve with reaction progress, including evolution of the catalytic volcano plot, evolution of the apparent enthalpy profile, and evolution of the effective diffusion coefficient.

## 7 Conclusions

The dam-break effect in MgH_2_ dehydrogenation shows that gas–solid reaction kinetics cannot always be reduced to a single activation energy, equilibrium parameter, or single-stage rate expression. Reaction progress should instead be treated as a physical coordinate that describes how kinetic resistance, local structure, and rate-controlling events evolve during conversion. In MgH_2_, hydrogen removal from the first surface layer gives rise to the highest initiation barrier. Once this layer is breached, subsequent hydrogen migration and desorption become easier, and the macroscopic activation energy rapidly decreases before entering a plateau-like regime. This stage-resolved picture explains why onset dehydrogenation temperature, apparent activation energy, and theoretical barriers have often appeared inconsistent. These quantities are not inherently contradictory, but correspond to different segments of the same evolving reaction. The dam-break effect therefore links atomic-scale surface dehydrogenation with macroscopic kinetic evolution and reframes MgH_2_ dehydrogenation as a process of dynamic resistance redistribution.

This framework suggests that MgH_2_ should be studied and optimized according to reaction stage rather than single averaged kinetic parameters. Mechanistically, it shifts attention from isolated elementary steps to the evolving local environment in which these steps occur. From a data-driven perspective, future databases and machine learning models should distinguish the reaction stage being captured, enabling catalysts, alloying, nanostructuring, and processing strategies to be compared according to their effects on the onset, resistance-release, or plateau regions. MLPs and multiscale simulations can further connect static barriers, atomic trajectories, and experimental kinetic curves. For application, the key target is not simply lowering an average activation energy, but matching materials design and energy input to stage-dependent kinetic demand. More broadly, MgH_2_ dehydrogenation provides a model case for gas–solid reactions in which interfaces, defects, product layers, and local thermodynamic environments evolve with progress, offering a basis for stage-specific regulation of reaction rates.
